# Effects of the Endpoint Adjudication Process on the Results of a Randomised Controlled Trial: The ADVANCE Trial

**DOI:** 10.1371/journal.pone.0055807

**Published:** 2013-02-04

**Authors:** Jun Hata, Hisatomi Arima, Sophia Zoungas, Greg Fulcher, Carol Pollock, Mark Adams, John Watson, Rohina Joshi, Andre Pascal Kengne, Toshiharu Ninomiya, Craig Anderson, Mark Woodward, Anushka Patel, Giuseppe Mancia, Neil Poulter, Stephen MacMahon, John Chalmers, Bruce Neal, on behalf of the ADVANCE Collaborative Group

**Affiliations:** 1 The George Institute for Global Health, University of Sydney, Sydney, New South Wales, Australia; 2 School of Public Health, Monash University, Clayton, Victoria, Australia; 3 Royal North Shore Hospital, Sydney, New South Wales, Australia; 4 Royal Prince Alfred Hospital, Sydney, New South Wales, Australia; 5 Sydney Adventist Hospital Clinical School, Sydney Medical School, University of Sydney, Sydney, New South Wales, Australia; 6 University of Milan-Bicocca, Milan, Italy; 7 Imperial College, London, United Kingdom; University Paris Descartes, France

## Abstract

**Background:**

Endpoint adjudication committees (EPAC) are widely used in clinical trials. The aim of the present analysis is to assess the effects of the endpoint adjudication process on the main findings of the ADVANCE trial **(**Trial registration: ClinicalTrials.gov NCT00145925).

**Methods and Findings:**

The ADVANCE trial was a multicentre, 2×2 factorial randomised controlled trial of blood pressure lowering and intensive blood glucose control in 11140 patients with type 2 diabetes. Primary outcomes were major macrovascular (nonfatal myocardial infarction, nonfatal stroke and cardiovascular death) and microvascular (new or worsening nephropathy and retinopathy) events. Suspected primary outcomes were initially reported by the investigators at the 215 sites with subsequent adjudication by the EPAC. The EPAC also adjudicated upon potential events identified directly by ongoing screening of all reported events. Over a median follow-up of 5 years, the site investigators reported one or more primary outcomes among 2443 participants. After adjudication these events were confirmed for 2077 (85%) with 48 further events added through the EPAC-led database screening process. The estimated relative risk reductions (95% confidence intervals) in the primary outcome for the blood pressure lowering comparison were 8% (−1 to 15%) based on the investigator-reported events and 9% (0 to 17%) based on the EPAC-based events (*P* for homogeneity = 0.70). The corresponding findings for the glucose comparison were 8% (1 to 15%) and 10% (2% to 18%) (*P* for homogeneity = 0.60). The effect estimates were also highly comparable when studied separately for macrovascular events and microvascular events for both comparisons (all *P* for homogeneity>0.6).

**Conclusions:**

The endpoint adjudication process had no discernible impact on the main findings in ADVANCE. These data highlight the need for careful consideration of the likely impact of an EPAC on the findings and conclusions of clinical trials prior to their establishment.

## Introduction

Much attention is given to the diagnosis of outcomes in large-scale multicentre trials because achieving consistency of reporting is perceived as a major challenge [2]. Standardized definitions of outcomes and protocols for their assignment are routinely used, but the possibility of misclassification remains. Accordingly, the design of most recent large-scale trials has included an endpoint adjudication committee (EPAC) that is blinded to study treatments and is responsible for assuring the validity of diagnoses for main trial outcomes [3]–[11]. However, it remains uncertain whether the endpoint adjudication process really improves the precision and validity of the treatment effects reported.

The Action in Diabetes and Vascular Disease: Preterax and Diamicron Modified Release Controlled Evaluation (ADVANCE) trial **(**Trial registration: ClinicalTrials.gov NCT00145925) was a multicentre, 2x2 factorial randomised controlled trial in patients with type 2 diabetes, which demonstrated separately beneficial effects of blood pressure lowering and intensive blood glucose control on the development of major macrovascular and microvascular diseases [1], [12], [13]. The aim of the present analysis is to assess the impact of the endpoint adjudication process on the main findings of the ADVANCE trial.

## Methods

### Ethics statement

Approval for the ADVANCE trial was obtained from each centre's institutional review board, and all participants provided written informed consent. A full list of 215 centres that participated in the trial was published previously [1].

### Design of the ADVANCE trial

**Figure 1:**
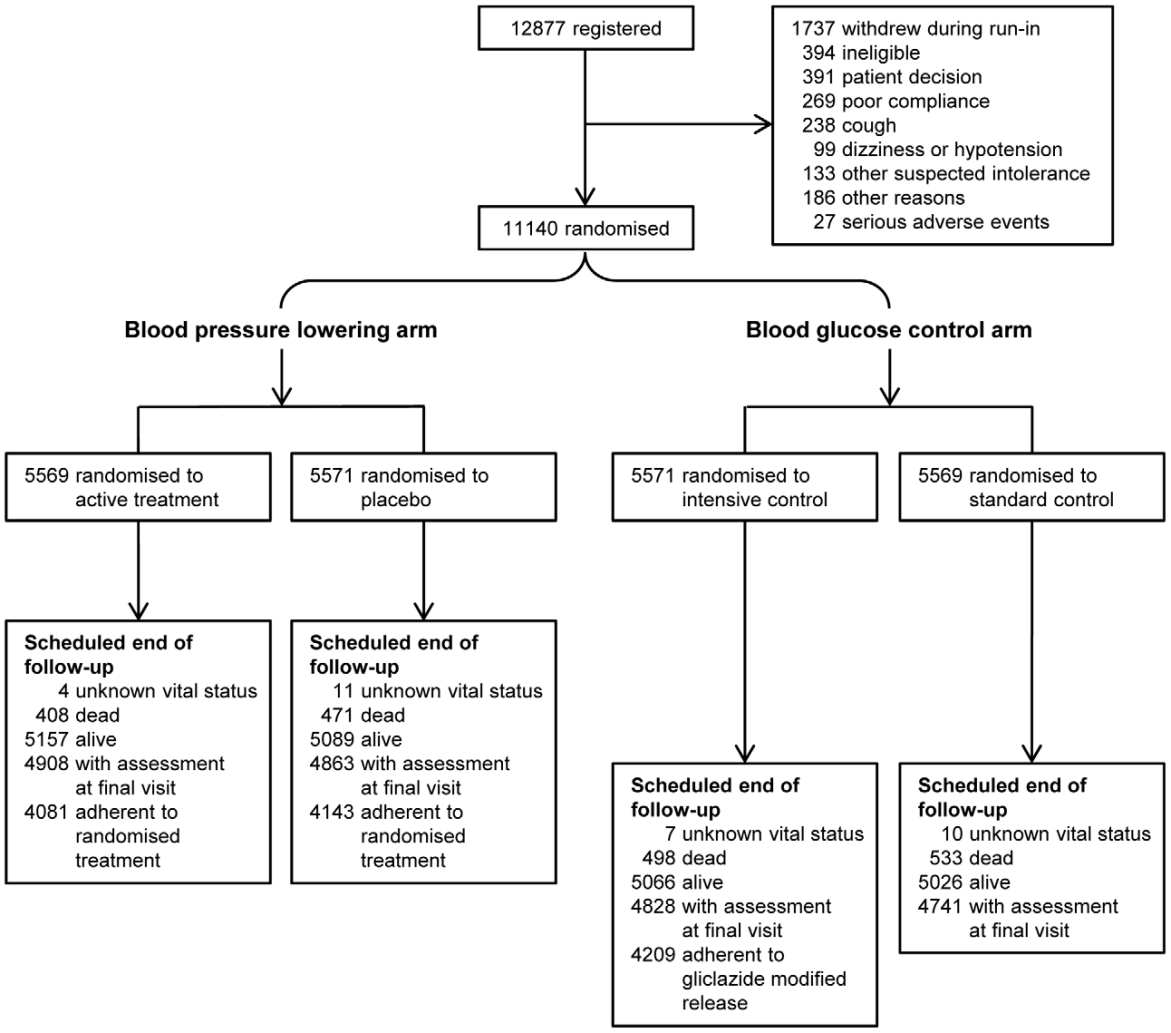
Enrolment, randomisation, and follow-up of study participants. A total of 11140 patients were randomly assigned, in a 2x2 factorial design, to active blood pressure lowering treatment with perindopril-indapamide or matching placebo, and to a gliclazide-based intensive glucose control strategy or a standard glucose control strategy.

The design of the ADVANCE trial has been described in detail previously [1], [12], [13], and the CONSORT checklist is available as Supporting Information, see Checklist S1. In brief, a total of 11140 patients with type 2 diabetes aged ≥55 years, with a history of major macrovascular or microvascular disease or at least one other risk factor for vascular disease, were enrolled from 215 centres in 20 countries between June 2001 and March 2003. After a 6-week active run-in period with fixed-combination of perindopril and indapamide during which usual glucose control was continued, participants were randomly assigned, in a 2x2 factorial design, to continued perindopril-indapamide or matching placebo and to either a gliclazide MR based intensive glucose control strategy aiming for a haemoglobin A1c of ≤6.5% or a standard glucose control strategy. Study treatments were allocated using a central, computer-based, randomisation service accessible by internet, telephone, and facsimile. Randomisation was stratified by study centre, history of macrovascular disease, history of microvascular disease, and background use of perindopril at baseline. All participants were allocated to one of the two randomised groups for both the blood pressure and the blood glucose interventions. The blood pressure lowering intervention was a placebo-controlled double-blind design, in which site investigators, patients and endpoint adjudicators were all blinded to randomised treatment allocation. The blood glucose control intervention was a prospective randomised open blinded endpoint (PROBE) design, in which site investigators and patients were not blinded but all endpoint adjudicators were blinded. Median treatment follow-up was 4.3 years for the blood pressure lowering arm of the trial, and 5 years for the glucose control intervention (Figure 1).

### Definitions of study outcomes

The primary outcomes of the ADVANCE trial were a composite of major macrovascular events (nonfatal myocardial infarction, nonfatal stroke, or cardiovascular death) and a composite of major microvascular events (new or worsening nephropathy or retinopathy), considered both jointly and separately. Myocardial infarction was defined as the presence of any two of the following three criteria; (1) a history of typical ischaemic symptoms lasting for ≥15 minutes and unresponsive to sublingual nitrates (if given), (2) diagnostic electrocardiogram changes (e.g. ST segment elevation/depression, new pathological Q wave), and (3) raised biochemical markers of myocardial damage (e.g. creatine kinase, troponin T); or autopsy findings of acute myocardial infarction (International Classification of Diseases 10th Revision [ICD-10] codes of I21.0-I21.9, I22.0-I22.9). Stroke was defined as a clinical history of acute disturbance of focal neurological function resulting in symptoms lasting >24 hours and thought to be due to brain infarction or intracranial haemorrhage (ICD-10 codes of I61.0-I61.9, I62.1, I62.9, I63.0-I63.9, I64) supported by brain imaging or autopsy. Subarachnoid haemorrhage, subdural haemorrhage and transient ischaemic attack were not included in the definition of stroke. Nonfatal myocardial infarction or nonfatal stroke was defined as an event that did not result in death within 28 days from onset. Cardiovascular death was defined as any death in which the proximate or the underlying cause of death was due to a disease of the circulatory system (ICD-10 codes of I10-I14, I20-I25, I26, I27.9, I28, I50-I52, I60-I67, I69, I70-I79, I80-I89) or a sudden death (ICD-10 codes of R96.0, R96.1, I46.1, R98). New or worsening nephropathy was defined as development of macroalbuminuria (a urinary albumin to creatinine ratio >300 µg/mg) confirmed by two positive results, doubling of the serum creatinine to a level of at least 200 µmol/L, the need for renal replacement therapy (dialysis or transplantation), or death from renal disease (ICD-10 codes of N00-N29, E11.2, I12, I13). New or worsening retinopathy was defined as development of proliferative retinopathy, macular oedema or diabetes-related blindness, or the use of retinal photocoagulation therapy. A secondary outcome, also included in the present analysis, was death from any cause.

### Event reporting by the site investigators

The site investigators were a diverse group of physicians with different levels of experience trained in many different countries. Serious events recorded in the trial (including all suspected primary and secondary outcomes) were identified and first reported by one of the local site investigators using a standard “serious event form” accompanied by specified supporting documents. These outcomes were all assigned an ICD-10 or study-specific code (for those without applicable ICD-10 codes) by the clinical coordinator of the ADVANCE trial on the basis of the site investigators' diagnoses.

### Event reporting by the EPAC

All possible primary outcomes and deaths (both cardiovascular and noncardiovascular) were reviewed by the EPAC whose members (comprising cardiologists, neurologists, endocrinologists, nephrologists and ophthalmologists) were blinded to randomised treatment assignments. Using the supporting documents (e.g. electrocardiogram findings, laboratory test reports, findings of brain computed tomography or magnetic resonance imaging, clinical notes, ophthalmology reports, autopsy reports and death certificates), which had been translated as necessary, the EPAC either confirmed or refuted the initial diagnosis reported by the site investigators using standardized definitions and protocol. When the initial diagnosis made by the site investigator was refuted, an alternative diagnosis was provided and an ICD-10 or study-specific code assigned. In addition, throughout the trial we conducted central searches of all information collected on serious events and follow-up assessments, to identify possible primary outcomes that might have been incorrectly or incompletely reported by the site investigators. For these potential additional events supporting data were sought from the site investigators and then reviewed by the EPAC in the same way. Consequently, the EPAC identified misclassification in the site investigators' diagnosis (e.g. misclassification between myocardial infarction and stroke, misclassification between fatal and nonfatal myocardial infarction or stroke, incorrect causes of death). The EPAC also reviewed incomplete or unclassified events (e.g. unspecified cardiovascular disease, death of unknown cause) and determined whether these events met the definition of each outcome or not. In addition, laboratory data such as serum creatinine and urine albumin/creatinine ratio in the follow-up assessments were reviewed to identify possible unreported nephropathy events. Consequently, 3 events of nonfatal myocardial infarction, 4 of nonfatal stroke, and 10 of new or worsening nephropathy which were unreported or misclassified by the site investigators, were included into the outcomes by the EPAC. The EPAC also added 95 cardiovascular death events from the patients who were classified as death from non-cardiovascular or unknown causes by site investigators (Table 1).

**Table 1:** Number of events reported by the site investigators (SI) and confirmed, declined or added by the endpoint adjudication committee (EPAC) over a median follow-up of 5 years.

Outcome	Reported by SI	Confirmed by EPAC*	Declined by EPAC*	Added by EPAC†	Diagnosed by EPAC
Combined major macrovascular and microvascular events	2443	2077 (85.0%)	366 (15.0%)	48 (2.3%)	2125
Major macrovascular events	1310	1098 (83.8%)	212 (16.2%)	49 (4.3%)	1147
Nonfatal myocardial infarction	399	306 (76.7%)	93 (23.3%)	3 (1.0%)	309
Nonfatal stroke	576	419 (72.7%)	157 (27.3%)	4 (0.9%)	423
Cardiovascular death	487	447 (91.8%)	40 (8.2%)	95 (17.5%)	542
Major microvascular events	1357	1122 (82.7%)	235 (17.3%)	9 (0.8%)	1131
New or worsening nephropathy	606	512 (84.5%)	94 (15.5%)	10 (1.9%)	522
New or worsening retinopathy	852	681 (79.9%)	171 (20.1%)	0 (0.0%)	681
Death from any cause	1031	1031 (100.0%)	0 (0.0%)	0 (0.0%)	1031

* Number and percentage among the events originally reported by SI.

† Number and percentage among the events finally diagnosed by EPAC.

### Statistical methods

The impact of the endpoint adjudication process on the main results of the ADVANCE trial was estimated by calculating the treatment effects on the outcomes of interest using univariate Cox proportional hazards models. The models were first fitted using the dataset based on the site investigators' initial diagnoses and second using the dataset based on the EPAC final diagnoses. For composite outcomes and patients with multiple events, the first applicable event was used in each analysis. All analyses were done according to the principle of intention to treat with relative risk reductions reported as percentage reductions ([1 - hazard ratio] ×100). The homogeneity between the treatment effects estimated using the site investigators' diagnoses and the EPAC diagnoses was addressed through a test of the null hypothesis that the EPAC hazard ratio was equal to the investigators hazard ratio for each outcome [3]. This test exploits the sequential nature of the diagnostic process; the EPAC diagnosis was made in the knowledge of the investigators' diagnosis. In addition, we estimated the percentage of error saved by the endpoint adjudication process through a comparison of mean square errors (MSEs) [3]. Assuming that the effect estimate based on the EPAC diagnoses is unbiased, the percentage of error saved by the endpoint adjudication process was estimated as  = {(√MSE[i] -√MSE[e])/√MSE[i]}×100, where MSE[e] is the variance of the log hazard ratio based on the EPAC diagnoses and MSE[i] is the variance of the log hazard ratio based on the investigators' diagnoses plus the squared difference between the log hazard ratio estimate based on the investigators' and the EPAC diagnoses. The bias-corrected and accelerated mean and 95% confidence interval (CI) of the percentage of error saved by the endpoint adjudication process (which had a skewed distribution) was estimated from 10,000 bootstrap samples. All analyses were performed using SAS 9.2 (SAS Institute, Cary, North Carolina, USA).

## Results

### Comparison of diagnoses made by site investigators and the EPAC

Over a median follow-up of 5 years, the site investigators reported one or more primary macrovascular or microvascular outcome for 2443 participants. After adjudication these events were confirmed for 2077 (85%) with 48 further events added through the database screening process (Table 1). The numbers of events initially reported by the site investigators and finally diagnosed by the EPAC were 1310 and 1147 for major macrovascular events, 1357 and 1131 for major microvascular events, and 1031 and 1031 for death from any cause, respectively. The proportion of the investigator-reported events confirmed by the EPAC was greater than 70% for all the composite primary outcomes and their major components.

### Impact of endpoint adjudication on the effect estimates for blood pressure lowering comparison

**Figure 2:**
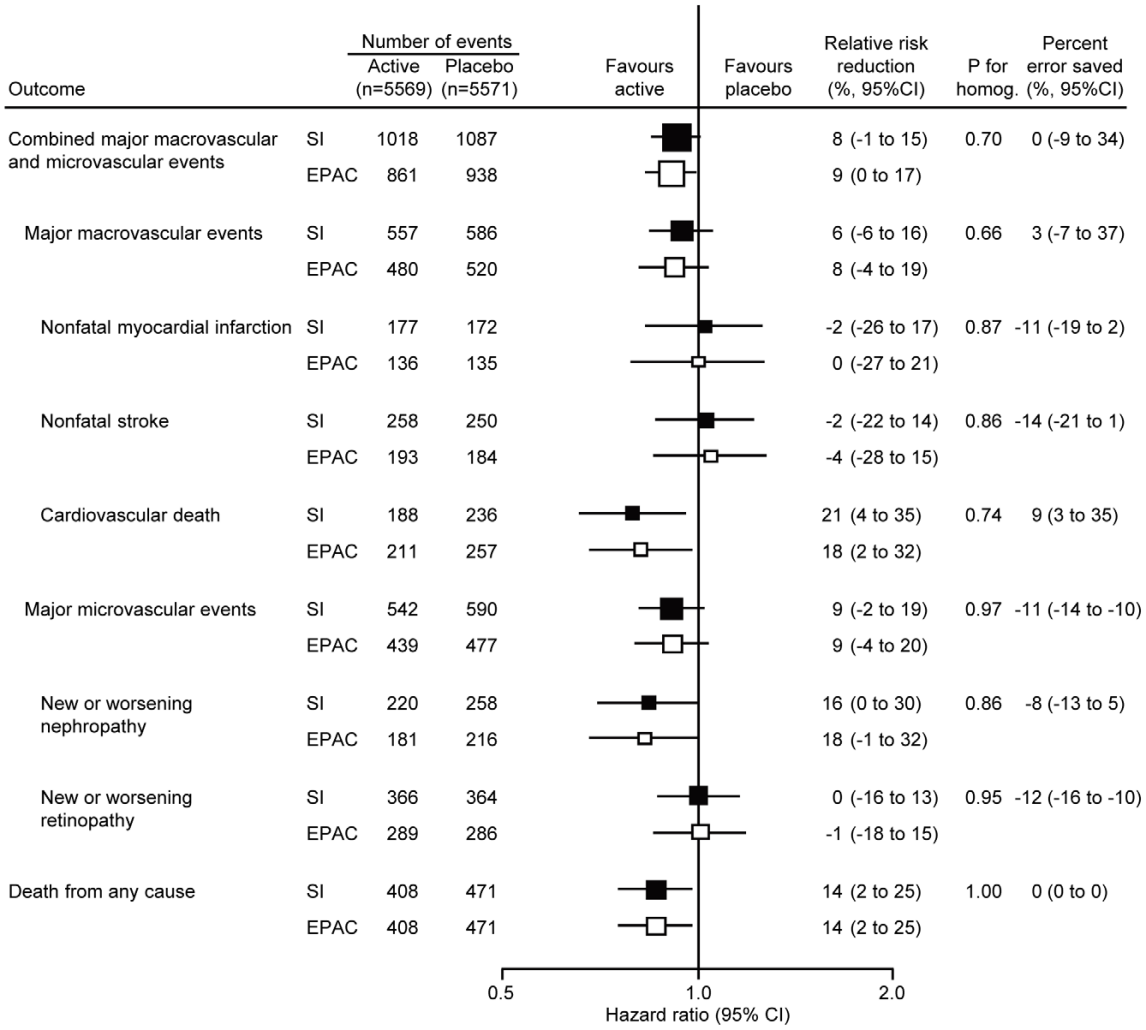
Effects of endpoint adjudication on the results of ADVANCE blood pressure lowering arm. Effects of blood pressure lowering treatment on the risks of clinical outcomes were examined based on diagnoses reported by the site investigators (SI) and those assigned by the endpoint adjudication committee (EPAC). Centers of the boxes are placed at the estimates of effect; areas of the boxes are proportional to the reciprocal of the variance of the estimates. Horizontal lines represent 95% confidence intervals (CI).

Figure 2 demonstrates the effects of blood pressure lowering treatment on the relative risk reductions of outcomes based on the initial diagnoses made by the site investigators and those based on the final diagnoses of the EPAC. Perindopril/indapamide-based blood pressure lowering treatment reduced the risk of combined macrovascular and microvascular events based on the investigators' diagnoses by 8% (95% CI -1 to 15%) and those based on the EPAC diagnoses by 9% (95% CI 0 to 17%) (*P* = 0.70 for homogeneity). Estimates of treatment effect based on the investigators' diagnoses and the EPAC diagnoses were highly comparable for every other outcome reported for this comparison (all *P* for homogeneity>0.6). In addition the estimated percent error saved by the adjudication process was small in every case.

### Impact of endpoint adjudication on the effect estimates for the glucose comparison

**Figure 3:**
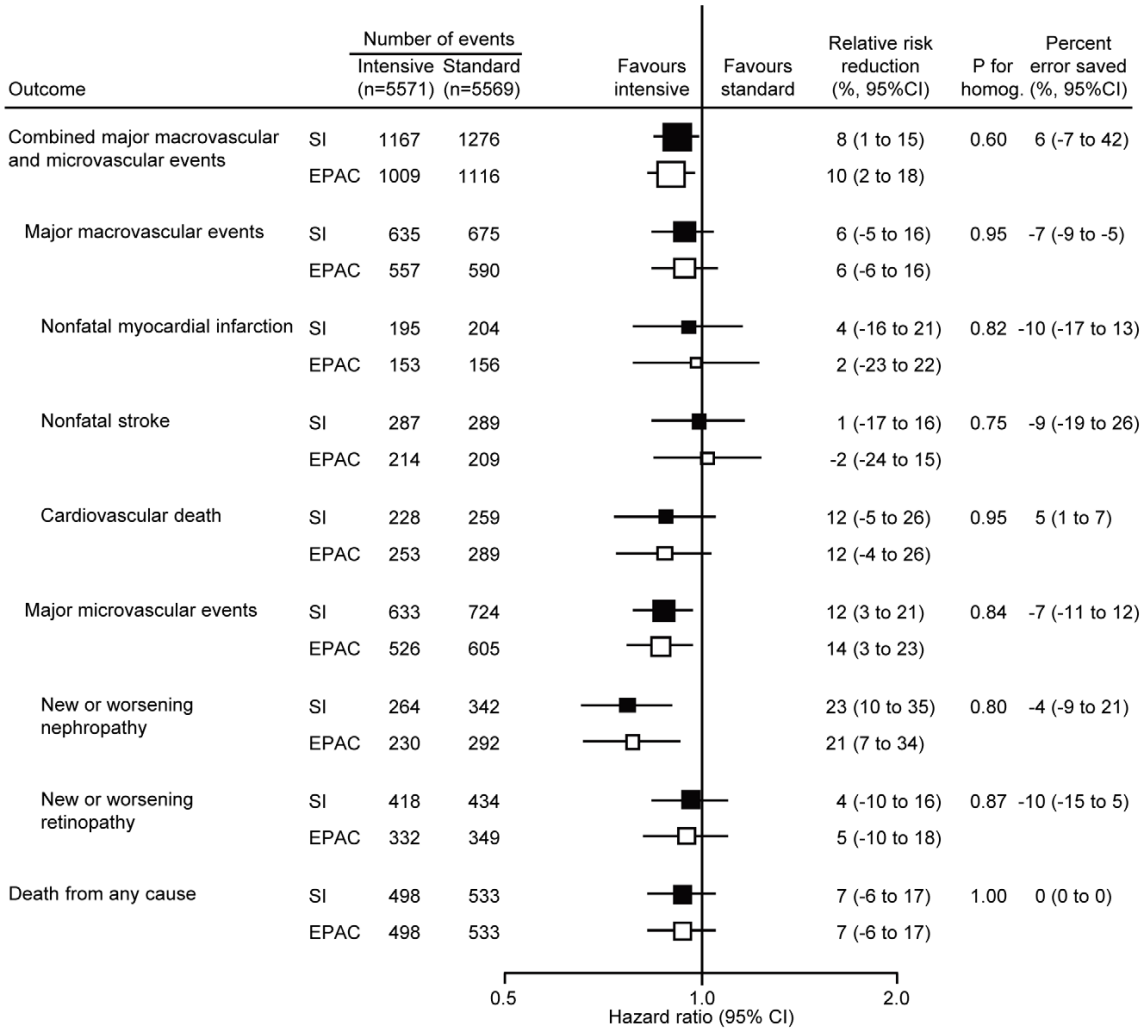
Effects of endpoint adjudication on the results of ADVANCE blood glucose control arm. Effects of intensive glucose control on the risks of clinical outcomes were examined based on diagnoses reported by the site investigators (SI) and those assigned by the endpoint adjudication committee (EPAC). Centers of the boxes are placed at the estimates of effect; areas of the boxes are proportional to the reciprocal of the variance of the estimates. Horizontal lines represent 95% confidence intervals (CI).

The effects of intensive glucose control on relative risks are shown in Figure 3. There were comparable reductions in the relative risks of combined macrovascular and microvascular events based on the investigators' diagnoses (8%, 95% CI 1 to 15%) and on the EPAC diagnoses (10%, 95% CI 2 to 18%) (*P* for homogeneity = 0.60). Likewise, there was no difference in estimates of treatment effect based on the investigators' diagnoses and the EPAC diagnoses for every other outcome (all *P* for homogeneity>0.7) and the percent error saved by the adjudication process was again minimal in every case.

## Discussion

In the present analysis, the endpoint adjudication process moved the point estimates of relative risk reductions in the blood pressure arm slightly to the left (more favourable in the active group) for 4 outcomes, slightly to the right (less favourable) for 3 outcomes, and left the estimate unchanged for 2 outcomes. The corresponding 95% CIs were made slightly wider by adjudication for 7 outcomes, slightly narrower for 1 outcome and were unchanged for 1 outcome. In the glucose arm, point estimates were moved slightly to the left for 3 outcomes, slightly to the right for 3 outcomes, and unchanged for 3 outcomes; and 95% CIs were made slightly wider for 7 outcomes, slightly narrower for 1 outcome and were unchanged for 1 outcome. For none of the analyses were the estimates based on the adjudicated outcomes shown to be substantially different from the estimates made using the original diagnoses reported by the investigators, and in every case the ‘error saved’ through the adjudication process was small. Our findings raise some uncertainty about the purely scientific value of the endpoint adjudication process in large-scale randomised controlled trials with characteristics similar to those of the ADVANCE trial, though as discussed below this may vary with trial design. On the other hand, the adjudication process does provide reassurance to users of the trial results including health funders and regulators. While the scientific value of endpoint adjudication in large-scale randomised controlled trials may be debatable, it is clear that it will remain an essential element for trial design as long as regulatory agencies insist upon it before approving new treatments or devices.

Multicentre trials are clearly at risk of outcome misclassification caused by differential application of definitions by site investigators. Three factors seem to be associated with the risk of misclassification and the necessity of adjudication processes; namely the nature of outcomes, the quality of site investigators and the study design.

The nature of the outcomes is a primary factor which is associated with the risk of misclassification. This risk can be minimized by adopting ‘hard outcomes’ with clear and objective definitions. We recently reported that the misclassification of outcomes in the Perindopril Protection Against Recurrent Stroke Study (PROGRESS) (i.e. stroke, myocardial infarction and cause-specific death) is infrequent and that the EPAC had little impact on treatment effects [3]. A recent systematic review of investigator and EPAC diagnoses showed good inter-observer agreement for reporting of macrovascular outcomes of this type [4]. In the present study, we reconfirmed similar results for macrovascular outcomes and death. In addition, the present study is the first report to confirm the lack of significant influence of the adjudication process on the estimates of microvascular complications in diabetes. The macrovascular and microvascular events that formed the primary outcome in ADVANCE are fairly easy to diagnose correctly and death is the least likely event to be misreported. On the other hand, there will be some circumstances in which adjudication is required. For example, in the Platelet Glycoprotein IIb/IIIa in Unstable Angina: Receptor Suppression Using Integrilin Therapy (PURSUIT) trial, in patients with acute coronary syndromes (ACS), importantly different estimates of treatment effects have been reported based on adjudicated and unadjudicated diagnoses of myocardial infarction [5], [6]. Although myocardial infarction is usually considered to be a hard outcome and easy to diagnose (as in the ADVANCE trial), the accurate diagnosis of myocardial infarction may be more difficult in specific circumstances, such as the acute setting of ACS in the PURSUIT trial. Thus, in studies using ‘soft outcomes’ which are difficult to diagnose objectively or derived from less reliable sources, the adjudication process may exert a significant impact on the study conclusions.

The quality of site investigators may be also an important factor. In ADVANCE, the sites involved were centres of excellence in their respective countries with leadership by senior physicians in the field. Large scale clinical trials have rigorous site selection processes and it may well be that careful site selection is able to provide similar degrees of assurance of reliable outcome reporting to that obtained through a separate endpoint adjudication process.

Study design is possibly the most important determinant of the necessity for an independent endpoint adjudication process. Even if hard outcomes and careful site selection do apply, it is impossible to eradicate outcome misclassification completely. However, this need not be a major issue provided rigorous randomization is used, thus ensuring that the risks of misclassification are nondifferential between the treatment groups to be compared. Such nondifferential misclassification can be achieved in double-blinded randomised trials (e.g. blood pressure arm in ADVANCE) in which site investigators report events without any knowledge of study treatments. If the misclassification is infrequent and nondifferential, adjudication processes may have little impact on point estimates of treatment effect. The risks of differential misclassification are theoretically greater in open-label trials, where site investigators may have preconceived notions about the treatment effect and may report outcome-related events differentially between the treatment groups. Even here however, these risks may be minimized through use of a PROBE design with reliance on a blinded adjudication process, as was used in the glucose arm of ADVANCE. An adjudication process may also be important for observational studies in which differential misclassification between the study groups may be more likely.

In the present analysis in ADVANCE, the systematic slight widening of the CIs in relative risks in both treatment arms was attributable to the EPAC discarding a small number of the events originally reported by the site investigators. There was no evidence of bias in the point estimates of treatment effect based on unadjudicated outcomes. Therefore, it would appear that the primary impact of the adjudication process was to slightly reduce the statistical power.

Endpoint adjudication in the ADVANCE trial consumed very considerable resources and, although a formal estimate of cost was not possible, likely required more than a million dollars. These resources were mostly expended on the collection of additional data from sites, translation of documents, payment of the adjudicators and central coordination including the establishment of a dedicated database and tracking tool. With clinical research costs escalating, it is the responsibility of researchers to ensure that scarce resources are applied as sparingly and as efficiently as possible.

In conclusion, the endpoint adjudication process used in the ADVANCE trial had no discernible effect on the main findings of the trial in regard to either macrovascular or microvascular outcomes. These data highlight the need for careful consideration of the likely impact of an EPAC on the findings and conclusions of clinical trials prior to their establishment. The appointment of an EPAC has now become a knee jerk response in the design of large-scale clinical trials, but actually warrants the same careful scientific consideration as other aspects of the trial design. Thus the need for formal endpoint adjudication may vary with the trial design, the outcomes and the settings in which the particular trial is conducted. Formal quantitative estimates of the likelihood of an adjudication process influencing trial conclusions might also be used to better understand the potential benefits of implementing an EPAC. In addition, national and international regulatory agencies could play a lead role in rationalizing the use of adjudication processes by providing explicit advice based on a clear understanding of what an EPAC can reasonably be expected to contribute. Although the reassurance that the EPAC provided to the users of the ADVANCE trial was no doubt of substantial importance, there may be more cost-efficient ways of achieving this goal.

## Supporting Information

Checklist S1 **CONSORT checklist of the present study.**
(DOC)
